# Peptide adjacent to glycosylation sites impacts immunogenicity of glycoconjugate vaccine

**DOI:** 10.18632/oncotarget.19944

**Published:** 2017-06-30

**Authors:** Zhongrui Ma, Huajie Zhang, Peng George Wang, Xian-Wei Liu, Min Chen

**Affiliations:** ^1^ The State Key Laboratory of Microbial Technology, National Glycoengineering Research Center, School of Life Sciences, Shandong University, Jinan, Shandong, China; ^2^ Department of Chemistry, Georgia State University, Atlanta, Georgia, United States

**Keywords:** glycoconjugate vaccine, E. coli N-glycosylation system, glycosylation site, immunogenicity, glycopeptide, Immunology

## Abstract

Glycoconjugate vaccine is composed of polysaccharides (PSs) covalently linked with carrier protein. Glycosylation site selection, as a significant factor leading to heterogeneities of glycoconjugate structure, draws more and more attentions for its impact on the immunogenicity of glycoconjugate vaccine. To elucidate the relationship between glycosylation connectivity and immunogenicity of glycoconjugate vaccine, in this study, anti-*E. coli* O157:H7 glycoconjugate O-PS-MBP with defined connectivity, and three selected peptide segments GS1, GS2, GS3 derived from O-PS-MBP was synthesized. Immunogenicity results showed that only peptides adjacent to the glycosylation sites (GS1 and GS2) promoted the generation of PS-specific IgG antibodies and contributed to PS-specific IgG subclass distribution. Furthermore, GS1 and GS2 had significant priming effect for eliciting PS-specific IgG antibodies. These results indicated that different locations of glycosylation sites could lead to diverse presentation of peptides and glycopeptides to APCs and influence the immunogenicity of glycoconjugate vaccine, which extend the current understanding of mechanism for adaptive immune system activation by glycoconjugate vaccine, and have implications for rational glycoconjugate vaccine design.

## INTRODUCTION

Glycoconjugate vaccine, composed of polysaccharides (PSs) covalently linked with carrier protein, can significantly elicit PS-specific antibodies especially IgG [1-4]. Recent studies have placed much emphasis on glycoconjugate modification to enhance its antibody response [5-11]. Glycosylation site selection, as a significant factor leading to heterogeneities of glycoconjugate structure, draws more and more attentions for its impact on the immunogenicity of glycoconjugate vaccine [9, 12].

Recent studies have revealed that glycoconjugate is trimmed into peptides and glycopeptides in antigen presenting cells (APCs). Herein, peptides bind to major histocompatibility complex II (MHCII) and then are presented to T cells, glycopeptides bind to MHCII via their peptide epitopes and then their PS epitopes are presented to T cells [9]. It is reasonable to deduct that different locations of glycosylation sites could lead to diverse presentation of peptides and glycopeptides to APCs and then to be presented to T cells, which might influence the immunogenicity of glycoconjugate vaccine.

To elucidate the relationship between glycosylation connectivity and immunogenicity of glycoconjugate vaccine, in this study, anti-*E. coli* O157:H7 glycoconjugate O-PS-MBP with defined connectivity, and three selected peptide segments derived from O-PS-MBP were synthesized. In terms of their immunogenic properties in BALB/c mice, we explored different roles of peptides and glycopeptides trimmed from O-PS-MBP on the magnitude and breadth of PS-specific IgG antibodies.

## RESULTS

### Synthesis of glycoconjugate O-PS-MBP with defined glycosylation sites

**Figure 1:**
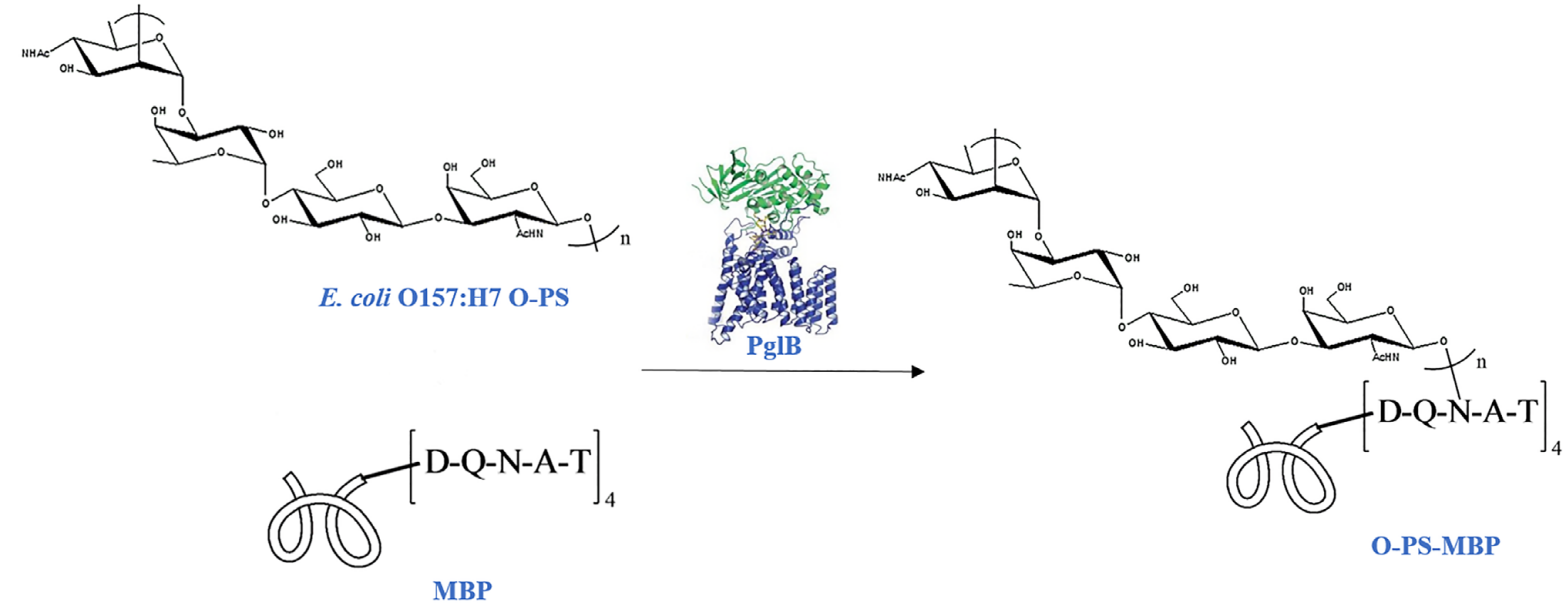
Schematic for biosynthesis of O-PS-MBP In *E. coli* CLM24 periplasm, transmembrane protein PglB catalyzes the conjugation of *E. coli* O157:H7 O-PS with the reducing end GalNAc to the asparagine residue amino acid sequence (DQNAT)_4_ within carrier protein MBP.

To distinguish peptides and glycopeptides generated from glycoconjugate, one simple way is using glycosylation site-specific glycoconjugate. Site-selective chemical conjugation methods have been emerging in recent years, but are almost largely empirical process with high production cost [13-18]. In this study, we investigated the utility of protein glycan coupling technology (PGCT) to produce homogenic glycoconjugate via one-shot *in vivo E. coli* fermentation [19]. *Campylobacter jejuni (C. jejuni)* oligosaccharyltransferase PglB, the key enzyme involved in PGCT, is able to transfer PSs to the asparagine residue of the amino acid sequence D/E-X-N-Y-S/T (X, Y≠P) within carrier protein [19, 20]. In this study, maltose-binding protein (MBP) was selected as the carrier protein, because of its defined structure and well-proven immunological enhancement in our previous findings [21]. MBP was modified with a C-terminal peptide sequence containing four consecutive DQNAT motifs, reported as the optimal acceptor sequence recognized by PglB [22]. Then, the modified MBP together with PglB and *E. coli* O157:H7 O-PS were co-expressed in *E. coli* strain CLM24, to synthesize O-PS-MBP *in vivo* (Figure 1 and Supplementary Figure 1) [21].

### IgG antibodies generated by O-PS-MBP

BALB/c mice were immunized with O-PS-MBP with two booster injections on days 14 and 28. On day 35, sera were collected and titrated against O-PS and MBP respectively in ELISA assays (Table 1). The results showed an average anti-O-PS IgG titer of 4.37 and an average anti-MBP IgG titer of 5.84. When compared the anti-MBP IgG titers between MBP and O-PS-MBP immunized mice, O-PS had slight interference on the immunogenicity of O-PS-MBP, even with no significant difference (Table 1, *P* > 0.05), which could be explained by an average anti-MBP response which masks the contribution of O-PS on individual peptide segments of O-PS-MBP.

**Table 1:** Mean titers of serum anti-O-PS and anti-MBP antibodies elicited in mice by injection of MBP and O-PS-MBP.

Conjugate	anti-O-PS				anti-MBP			
	IgG	IgG1	IgG2a	IgG2b	IgG	IgG1	IgG2a	IgG2b
MBP	3.66±0.28	2.83±0.57	2.94±0.19	2.68±0.48	6.03±0.22	6.18±0.34	5.09±0.42	4.63±0.27
O-PS-MBP	4.37±0.38	3.99±0.32	4.48±0.14	4.00±0.51	5.84±0.42	5.91±0.16	4.90±0.32	4.54±0.24

NOTE. The antibody titer was shown as log_10_(cutoff value). The cutoff value was OD_negative control_×2.1.

### O-PS shows interference on anti-peptide IgG

**Figure 2:**
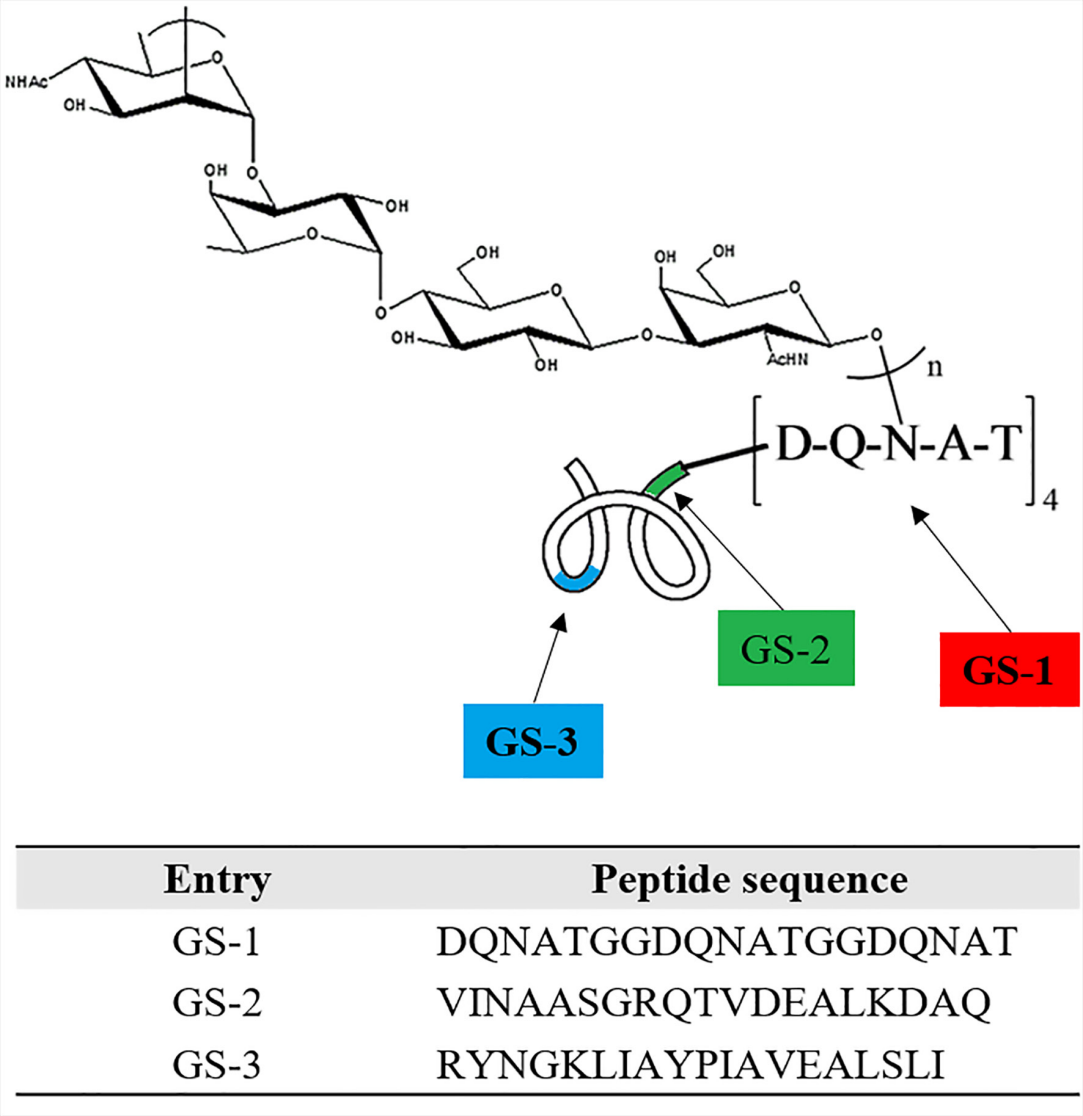
Schematic for peptides GS1, GS2 and GS3 GS-1: contains the glycosylation sites. GS-2: adjacent to the glycosylation sites with 2 amino acid space from (DQNAT)_4._ GS-3: remote from the glycosylation sites with 251 amino acid space from (DQNAT)_4_. See amino acid sequence of protein carrier MBP in Supporting Information for specific sequence and location details.

**Figure 3:**
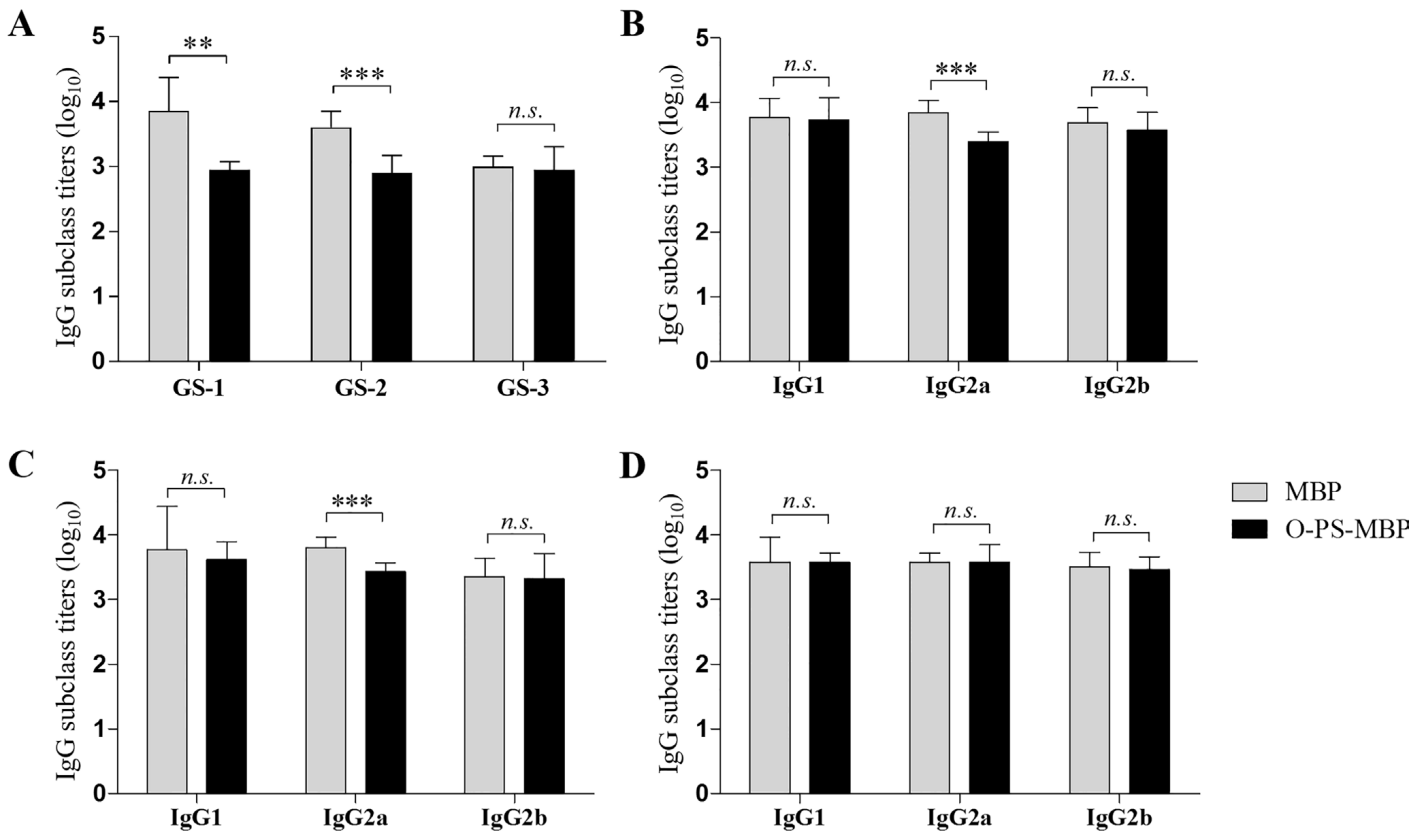
Anti-peptide IgG and subclass titers **A**. Anti-peptide IgG titers. **B**. Anti-GS-1 IgG subclass titers. **C**. Anti-GS-2 IgG subclass titers. **D**. Anti-GS-3 IgG subclass titers. Differences were indicated with symbols (**: *P* < 0.01; ***: *P* < 0.001; *n.s*.: *P* > 0.05). Results were expressed as the arithmetic mean ±SD indicated by error bars.

To further investigate the interference of O-PS on peptides at different locations of O-PS-MBP, three nonadeca peptide segments GS-1, GS-2 and GS-3 from O-PS-MBP were synthesized, herein, GS-1 containing the glycosylation sites, GS-2 adjacent to the glycosylation sites and GS-3 far from the glycosylation sites (Figure 2 and Supplementary Figure 2). In other words, GS-1 and GS-2 contained peptide epitopes of glycopeptides, while GS-3 contained peptide epitopes of vacant peptide sequences. The anti-GS-1, GS-2, GS-3 IgG titers in mice immunized with O-PS-MBP and MBP were evaluated, respectively (Figure 3A). The results showed that anti-GS-1 and GS-2 IgG titers decreased significantly when MBP was linked with O-PS (*P* < 0.001), whereas anti-GS-3 IgG response in immunization of O-PS-MBP had no significant difference compared with that of MBP, reflecting that O-PS blocked the generation of IgG antibodies against GS-1 and GS-2 but not GS-3. This phenomenon confirmed the mechanism that peptide epitopes of glycopeptides helped to present PS epitopes to T cells [9]. For that reason, the number of peptide epitopes themselves presenting to T cells reduced, which led to a decline of anti-peptide epitope IgG response.

As for the breadth of the IgG response, the anti-O-PS IgG was dominantly IgG2a (*P* < 0.05), but anti-MBP IgG was dominantly IgG1 (*P* < 0.0001). When compared the anti-MBP IgG subclass (IgG1, IgG2a, IgG2b) titers between MBP and O-PS-MBP immunized mice, O-PS had also slight interference on the immunogenicity of O-PS-MBP, even with no significant difference (Table 1, *P* > 0.05). When focusing on individual peptides, we found that anti-GS-1 and GS-2 IgG2a titers decreased dramatically when MBP was linked with O-PS (Figure 3B-D, *P* < 0.001), but not GS-3. These results can be explained that, due to GS-1 and GS-2 contained peptide epitopes of glycopeptides, the decline of anti-GS-1 and GS-2 IgG2a contributed to the increase of anti-O-PS IgG2a, then leading to that the anti-O-PS IgG was dominantly IgG2a. In other words, in glycopeptides, the IgG subclass profile against peptide epitope was consistent with that against O-PS.

### Peptide priming effect

**Figure 4:**
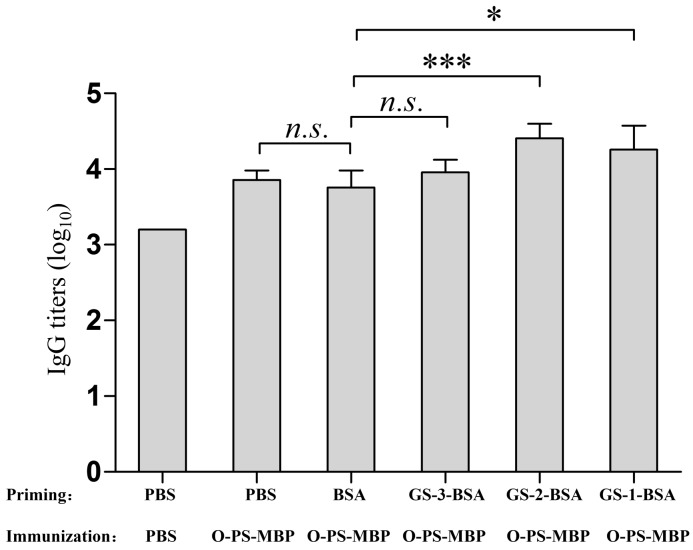
Anti-O-PS IgG titers Sera from priming with peptides and immunization of O-PS-MBP were tested for anti-O-PS IgG titers. Differences were indicated with symbols (*: *P* < 0.05; ***: *P* < 0.001; *n.s*.: *P* > 0.05). Results were expressed as the arithmetic mean ±SD indicated by error bars.

Inversely, to explore the impact of GS-1, GS-2, GS-3 on anti-O-PS IgG antibodies generated by O-PS-MBP, we primed the mice with GS-1-BSA, GS-2-BSA, GS-3-BSA (conjugating to a protein can enhance the immunogenicity of peptides), respectively, and then immunized the mice all with glycoconjugate O-PS-MBP. After the immunization, no significant difference was detected in anti-O-PS IgG titers between the mice primed with PBS and BSA, reflecting that BSA has no interference for eliciting anti-O-PS IgG titers by O-PS-MBP (Figure 4). Furthermore, the mice primed with GS-1-BSA or GS-2-BSA, rather than those primed with GS-3-BSA, induced higher anti-O-PS IgG titers compared with those primed with BSA (Figure 4). All above results showed that the peptide epitopes from glycopeptides had significant priming effects for eliciting PS-specific IgG titers. Only peptides at and near the glycosylation sites, not all peptides trimmed from carrier protein, had significant priming effects for eliciting PS-specific IgG titers by glycoconjugate vaccine.

## DISCUSSION

**Figure 5:**
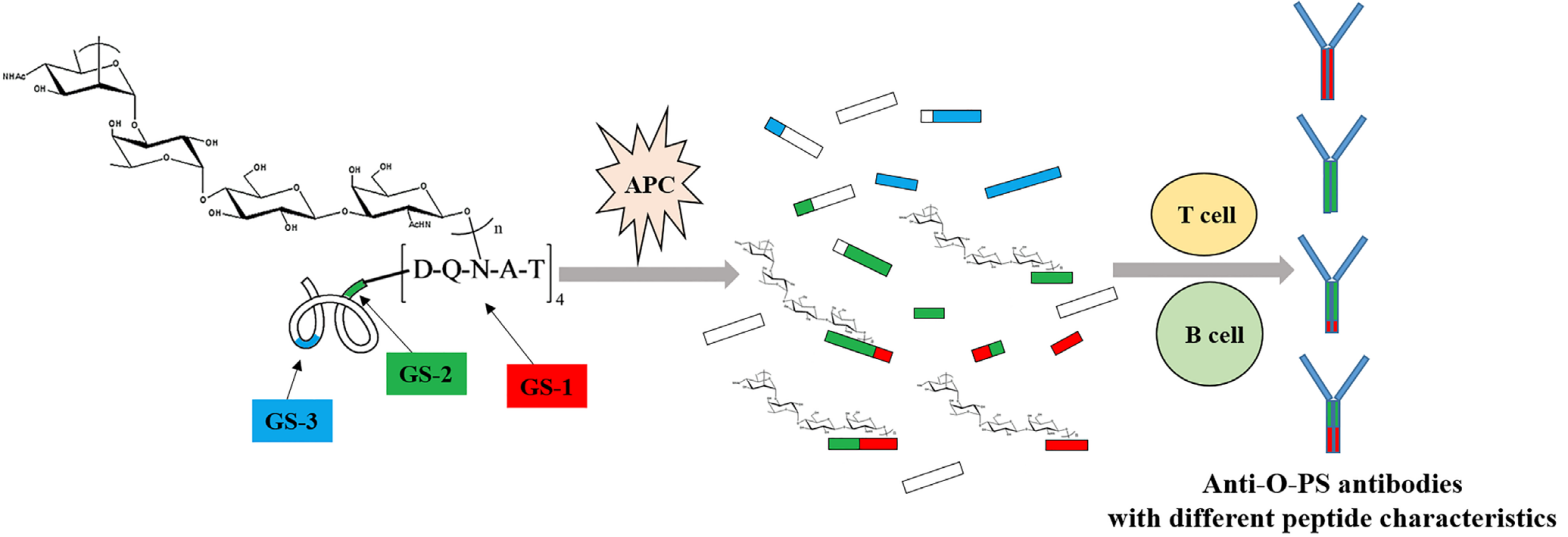
The proposed mechanism of adaptive immune system activation by glycoconjugate O-PS-MBP O-PS-MBP is trimmed into peptides and glycopeptides in APC. Herein, glycopeptides have two parts, peptide epitopes and PS epitopes. Those peptide epitopes help present PS epitopes to T and B cells to generate PS-specific antibodies. Correspondingly, peptide epitopes could not present themselves to T and B cells to generate peptide epitope-specific antibodies. Meanwhile, the immunogenicity properties of peptide epitopes are transferred to PS epitopes. In other words, the profile of generated PS-specific antibodies depends on the characteristics of peptide epitopes.

The importance of glycosylation site selection on the immunogenicity of glycoconjugate vaccine is not obvious because most of these glycoconjugates are made by random chemical methods. In this study, the immunogenicity results of glycoconjugate O-PS-MBP with defined glycosylation sites shows that glycopeptides rather than peptides trimmed from glycoconjugate have an important role in generating PS-specific IgG antibodies. Alternatively, the number and location of glycosylation sites could result in presenting of different glycopeptides to T cells and generating diverse PS-specific antibodies. Based on our findings, for an extension of recently proposed mechanism for adaptive immune system activation by glycoconjugate vaccine (mentioned in introduction), glycoconjugate is trimmed into peptides and glycopeptides in APC. Those peptide epitopes help present PS epitopes to T and B cells to generate PS-specific antibodies. Correspondingly, those peptide epitopes could not present themselves to T and B cells to generate peptide epitope-specific antibodies. Meanwhile, the immunogenicity properties of peptide epitopes are transferred to PS epitopes. In other words, the profile of generated PS-specific antibodies depends on the characteristics of peptide epitopes (Figure 5).

Carrier protein priming/suppression refers to improved/reduced PS-specific response to glycoconjugate vaccine in an individual previously immunized with carrier protein compared with whom not. It is well believed that priming with carrier protein enhances the PS-specific IgG response by increasing the number of protein-specific T cells, which help the proliferation and differentiation of PS-specific B cells [23, 24]. However, based on our findings, only peptides at and near the glycosylation sites, not all peptides trimmed from carrier protein, had significant priming effects for eliciting PS-specific IgG titers by glycoconjugate vaccines. This is a good explanation for a clinical phenomenon: high hapten density of the carrier protein results in priming. Reversely, low ratio of hapten to carrier protein results in suppression.

In conclusion, we have utilized a glycosylation site-selective conjugation method suitable for the synthesis of glycoconjugate O-PS-MBP, which allows a better investigation of the immunological mechanism of glycoconjugate vaccine. We found that the peptide epitopes from the glycopeptide fragments promote the generation of PS-specific IgG and apply their own subclass distribution properties on the PS-specific IgG subclass distribution. By altering the location of the glycosylation sites, the PS-specific IgG titer and subclass profile of glycoconjugate vaccine could be controlled.

## MATERIALS AND METHODS

### Ethics statement

This study was carried out in accordance with the recommendations of the Guide for the Care and Use of Laboratory Animals of the National Institutes of Health. The protocols were approved by the Ethics Committee of Shandong University School of Medicine (No. 001 in 2011 for Animal Ethics Approval) and all efforts were made to minimize sufferings.

### Bacterial strains, plasmids and growth conditions

*E. coli* DH5α was used for cloning of plasmids. *E. coli* CLM24 was used for glycoconjugate expression experiments. All *E. coli* strains were grown in Luria-Bertani (LB) broth. Ampicillin (100 μg/ml), chloramphenicol (25 μg/ml) and spectinomycin (50 μg/ml) were used for plasmids selection as needed. All strains and plasmids used in this study are listed in Supplementary Table 1.

### Construction of recombinant plasmids

The plasmid pYES1L-*E. coli* O157:H7 O-PS was constructed by inserting *E. coli* O157:H7 O antigen gene cluster and its upstream genes (including *wcaM, z3206, galF*) and downstream genes (including *gnd, ugd, wzz, hisl*) into plasmid pYES1L (GENEART^®^ High-Order Genetic Assembly System, Invitrogen)[21]. The plasmid pACT3-PglB was constructed by inserting *pglb* gene from *C. jejuni* NCTC 11168 into plasmid pACT3 at the *SmaI-Sal*I site. The plasmid pBAD24-MBP was constructed by inserting DNA encoding MBP, O-PS acceptor peptide tag GT (i.e., N-DQNATGGDQNATGGDQNATGGDQNAT-C) and a His6 tag (i.e., N-HHHHHH-C) into plasmid pBAD24 at the *SmaI-Sal*I site.

### Synthesis and characterization of peptides

The peptides GS-1, GS-2 and GS-3 were synthesized by Bankpeptide, Inc. China and detected by electrospray ionization-mass spectroscopy (ESI-MS) with a Shimadzu LCMS-IT-TOF 8030 spectrometer.

### BALB/c mice immunization

Six-week-old female BALB/c mice were injected subcutaneously with 30 μg (protein quantity) of O-PS-MBP or equivalent MBP per mouse, each group contained 8 mice. The mice were injected with three doses of 2 weeks’ interval. Six-week-old female BALB/c mice were injected subcutaneously with 30 μg (peptide quantity) of BSA conjugated peptides (GS-1-BSA, GS-2-BSA, GS-3-BSA) or controls (BSA, PBS) per mouse, each group contained 8 mice. The mice were injected with three doses of 2 weeks’ interval. Then, all groups of mice were injected subcutaneously with 30 μg (protein quantity) of O-PS-MBP per mouse. The mice were injected with two doses of 2 weeks’ interval. O-PS-MBP and MBP were formulated in PBS (pH 7.4) with Freund’s complete adjuvant (FCA) (Sigma) for the first immunization or Freund’s incomplete adjuvant (FIA) (Sigma) for the other immunizations. Seven days after the final immunization, the blood were taken from the tail vein of mice and centrifuged at 3,000g for 30 min to obtain sera.

### Analysis of IgG responses

The IgG responses against *E. coli* O157:H7 O-PS, MBP or peptides induced by different formulations, were measured by ELISA as described previously [21, 25]. Briefly, the 96-well plates (Costar^®^ Polystyrene High Binding Plate 3590) were coated with 100 μl of 1 μg/ml *E. coli* O157:H7 LPS in 0.05 M Na_2_CO_3_ (pH 9.8) or 10 μg/ml MBP in PBS (pH 7.4) or 10 μg/ml peptides (GS-1, GS-2, GS-3) in 0.05 M Na_2_CO_3_ (pH 9.8), followed by incubation at 4°C overnight. The coated plates were then washed with 250 μl PBS + Tween (PBST) (pH 7.4) for five times and then blocked with 2% (w/v) BSA in PBS for 2 h at room temperature. After washing as above, two-fold serial dilutions of sera in PBS were then added to each column. Plates were incubated for 2 h at room temperature and washed again. HRP-goat anti-mouse IgG, IgG1, IgG2a, IgG2b (Abcam) appropriately diluted in PBST were added and the plates were incubated for 1 h at room temperature. After another washing, 100 μl TMB solution was added to the plates and incubated for 15 min before 100 μl of 1 M HCl was added. The plates were read at OD_450_. Sera titers were expressed as the log10(maximal dilution fold) corresponding to a cut-off value at OD_negative control_×2.1.

### Statistical analysis

All figures and statistical analyses were generated using the program GraphPad Prism version 5.0. Data were shown as means ± standard deviation (SD). When two groups were compared, t test was used for analyses. One-way ANOVA was used to test for statistical significance of differences between three experimental groups.

## SUPPLEMENTARY MATERIALS FIGURES AND TABLE



## Abbreviations

PS: polysaccharide
APCs: antigen presenting cells
MHCII: major histocompatibility complex II
PGCT: protein glycan coupling technology
MBP: maltose-binding protein
LB: Luria-Bertani
FCA: Freund’s complete adjuvant
FIA: Freund’s incomplete adjuvant
SD: standard deviation
C. jejuni: *Campylobacter jejuni*
