# Supplementary material for: Peptide adjacent to glycosylation sites impacts immunogenicity of glycoconjugate vaccine
Source: Oncotarget. 2017 Jun 30;9(1):75–82. doi: 10.18632/oncotarget.19944 (PMC5787506; doi:10.18632/oncotarget.19944)
Supplement: Supplementary file 1 [file oncotarget-09-75-s001.pdf]

Peptide adjacent to glycosylation sites impacts immunogenicity of glycoconjugate vaccine

SUPPLEMENTARY MATERIALS

Expression and purification of glycoconjugate

E. coli CLM24 transferred with pACT3-PglB, pYES1L-O-PS and pBAD24-MBP was grown in 50 ml LB broth at 37°C for 16 h with shaking. Cultures were then inoculated 1/100 into 1 L LB broth and further grown at 37°C with shaking until OD_{600} reached 0.6. Subsequently, 0.1% (w/v) L-arabinose and 50 µM IPTG were added to induce the expression of PglB and MBP, respectively. After another incubation at 28°C for 6 h, 0.1% (w/v) L-arabinose was added again for further induction of MBP. After a total 20 h induction at 28°C, cells were harvested by centrifugation at 10,000 *g* for 10 min and the periplasmic component of the cells were extracted by lysozyme treatment (20 mM Tris-HCl (pH 7.5), 20% (w/v) sucrose, 1 mM EDTA, 1 mg/ml lysozyme, at 4°C for 1 h). Then, after centrifugation at 10,000 *g* for 30 min, cell debris was removed and the supernatant was loaded to a Ni-NTA column filled with 3 ml Ni-NTA agarose (GE Healthcare), which was pre-equilibrated with wash buffer (10 mM imidazole, 0.5 M NaCl, 20 mM Na_2HPO_4 / NaH_2PO_4 buffer, pH 7.4). Subsequently, the column was washed with wash buffer again and then eluted with elution buffer (250 mM imidazole, 0.5 M NaCl, 20 mM Na_2HPO_4 / NaH_2PO_4 buffer, pH 7.4). Fraction was collected and then desalted using centrifugal filter (Amicon® Ultra-15, Milipore) against PBS (PH 7.4).

Characterization of glycoconjugate

Samples were separated by 12% SDS-PAGE gels followed by coomassie staining or transferring onto polyvinylidene fluoride (PVDF) membranes. The PVDF membranes were probed with anti-His6 antibody (Beyotime, China) or *E. coli* O157:H7 antiserum (China Institute of Veterinary Drug Control Center). In case of anti-His6 antibody, HRP-rabbit anti-mouse IgG (Invitrogen) was used as the secondary antibody. In case of *E. coli* O157:H7 antiserum, HRP-goat anti-rabbit IgG (Invitrogen) was used as the secondary antibody. The mass of protein portion of glycoconjugate was measured using BCA assay (Beyotime, China). The mass of PS portion was measured using phenol-sulfate method as described previously [1, 2].

Extraction of *E. coli* O157:H7 LPS

E. coli O157:H7 LPS was extracted using the hot phenol-water method as previously described [3]. Briefly, 10 g dried *E. coli* O157:H7 cells were extracted with 100 ml 50% (v/v) aqueous phenol at 65°C for 20 min. After centrifugation at 10,000 *g* for 30 min, the cell debris was discarded and the top aqueous solution was dialyzed against deionized water to remove phenol. Then the solution was lyophilized and then dissolved in 10 ml deionized water. Subsequently, the solution was consecutively treated with DNase I, RNase A and Proteinase K (Thermo Scientific) according to the manufacturer's instructions. After ultracentrifugation at 110,000 *g* for 4 h, the precipitated gels were dissolved in deionized water and lyophilized to obtain pure LPS.

SEQUENCES

Amino acid sequence of protein carrier MBP

MKIKTGARILALSALTTMMFSASALAKIEEGKLVIWINGDKGYNGLAEVGKKFEKDTGI

KVTVEHPDKLEEKFPQVAATGDGPDIIFWAHDRFGGYAQSGLLAEITPDKAFQDKLYPF

TWDAVRYNGKLIAYPIAVEALSLIYNKDLLPNPPKTWEEIPALDKELKAKGKSALMFNL

QEPYFTWPLIAADGGYAFKYENGKYDIKDVGVDNAGAKAGLTFLVDLIKNKHMNADT

DYSIAEAAFNKGETAMTINGPWAWSNIDTSKVNYGVTVLPTFKGQPSKPFVGVLSAGIN

AASPNKELAKEFLENYLLTDEGLEAVNKDKPLGAVALKSYEEELVKDPRIAATMENAQ

KGEIMPNIPQMSAFWYAVRTAVINAASGRQTVDEALKDAQLE***DQNATGGDQNATGGD***

QNATGGDQNATVDHHHHHH

Herein, peptides ***GS-1***、GS-2、GS-3; ***Glycosylation sequence DT$_4$***; Signal peptide MalE; His6 tag.

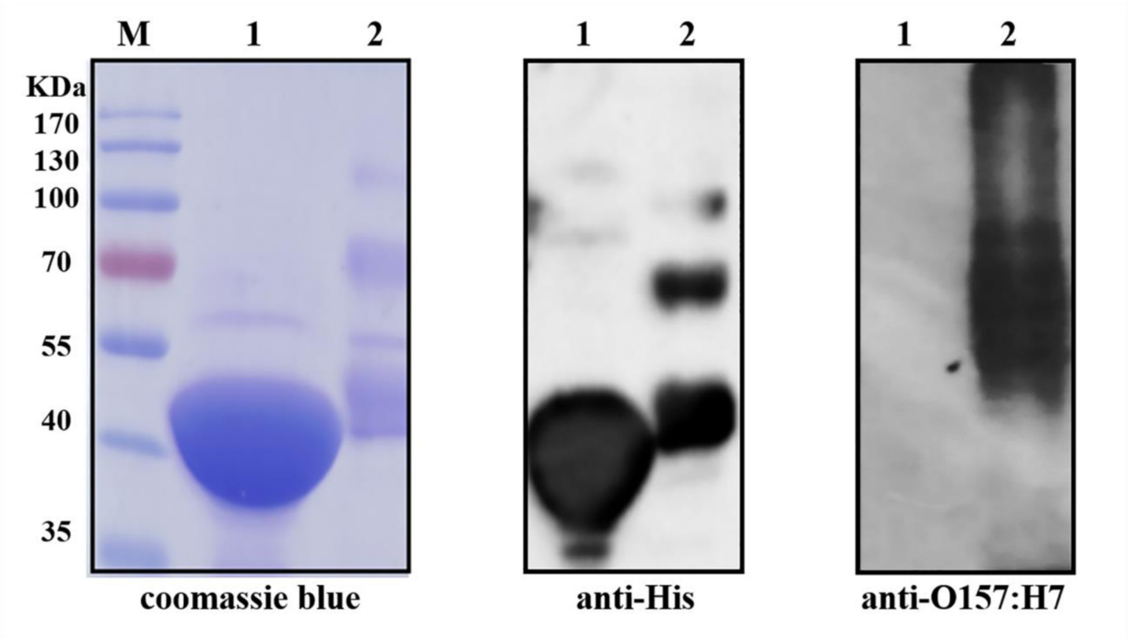

Supplementary Figure 1: Characterization of glycoconjugate O-PS-MBP. The samples from strain *E. coli* CLM24 carrying pACT3-PglB, pYES1L-O-PS, pBAD24-MBP was purified using Ni^{2+} affinity chromatography. After purification, samples were loaded onto a 12% SDS-PAGE gels and analyzed by coomassie blue staining and western blotting against anti-His6 antibody and *E. coli* O157:H7 antiserum. Strains of lane 1 were induced by L-arabinose, strains of lane 2 were induced by L-arabinose and IPTG. M: Protein maker.

A

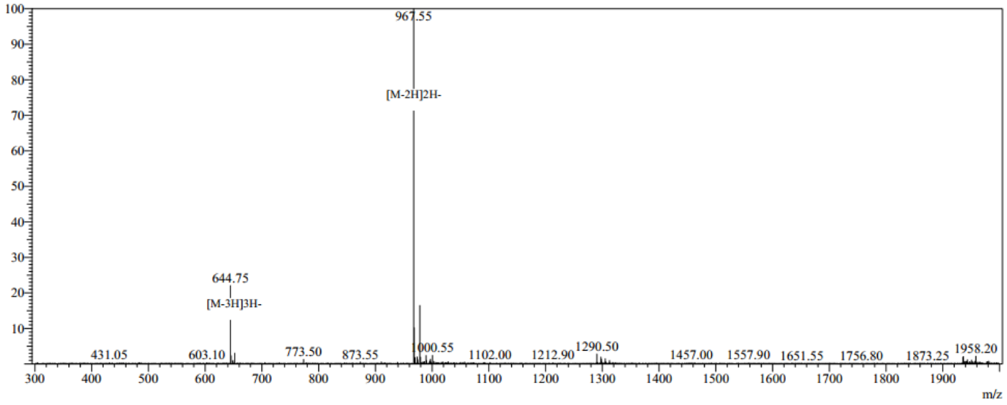

B

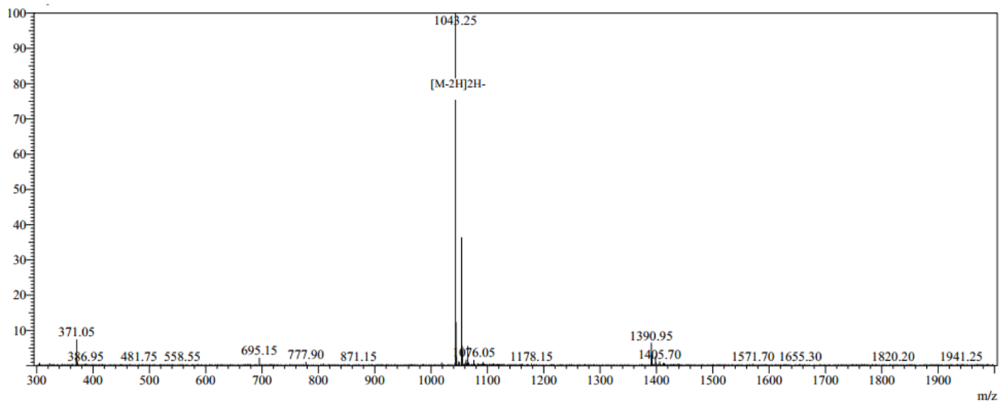

C

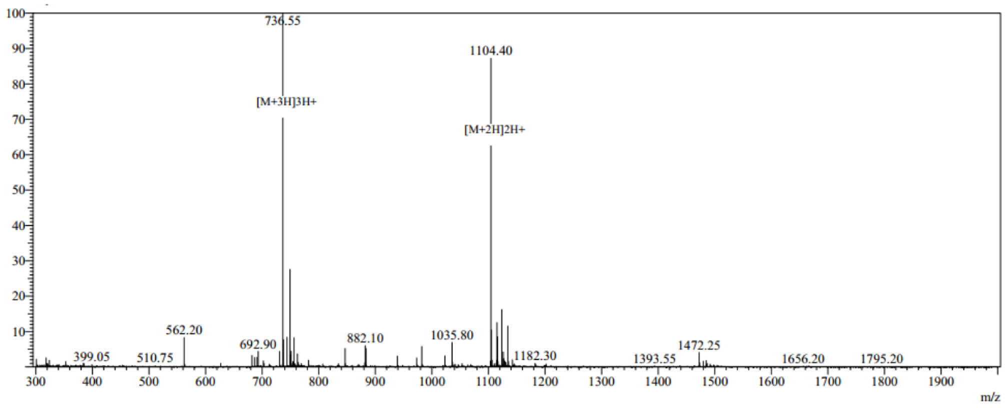

Supplementary Figure S2: MS analysis of peptides. A. GS-1. B. GS-2. C. GS-3.

Supplementary Table 1: Bacterial strains and plasmids used in this study.

Strain or plasmid	Description	Source
**Strain**		
E. coli DH5α	F- endA1 glnV44 thi-1 recA1 relA1 gyrA96 deoRnupG φ80d*lacZ*ΔM15 Δ(lacZYA-argF)U169, hsdR17($r_K^- m_K^+$), λ-	Novagen
E. coli W3110	F^- $λ^-$ rph-1 INV(rrnD, rrnE)	This study
E. coli CLM24	*E. coli* W3110 Δ*waal*	This study
**Plasmid**		
pBAD24	[a]Ap^r,araBAD promoter	[4]
pACT3	[b]Sp^r, *lacI*^{q}, *tac* promoter	[5]
pYES1L	[c]Cm^r	Invitrogen
pBAD24-MBP	Ap^r, pBAD24 containing *malE* gene	This study
pBAD24-CRM	Ap^r, pBAD24 containing *crm*$_{197}$ gene	This study
pBAD24-AcrA	Ap^r, pBAD24 containing *acrA* gene	This study
pACT3-PglB	Ap^r, pACT3 containing *pglb* gene	This study
pYES1L-O-PS	Cm^r, pYES1L containing *E. coli* O157:H7 O antigen *rfb* gene cluster	This study

REFERENCES

1. Iwashkiw JA, Fentabil MA, Faridmoayer A, Mills DC, Peppler M, Czibener C, Ciocchini AE, Comerci DJ, Ugalde JE, Feldman MF. Exploiting the Campylobacter jejuni protein glycosylation system for glycoengineering vaccines and diagnostic tools directed against brucellosis. Microb Cell Fact. 2012; 11: 13. doi: 10.1186/1475-2859-11-13.
2. Ma Z, Zhang H, Shang W, Zhu F, Han W, Zhao X, Han D, Wang PG, Chen M. Glycoconjugate vaccine containing Escherichia coli O157:H7 O-antigen linked with maltose-binding protein elicits humoral and cellular responses. PLoS One. 2014; 9: e105215. doi: 10.1371/journal.pone.0105215.
3. Mehl M, Starke R, Möckel C, Presber W. [Isolation of group-specific polysaccharide complexes from group B streptococci by phenol-water extraction]. [Article in German]. J Basic Microbiol. 1988; 28:437–44.
4. Guzman LM, Belin D, Carson MJ, Beckwith J. Tight regulation, modulation, and high-level expression by vectors containing the arabinose PBAD promoter. J Bacteriol. 1995; 177: 4121-30.
5. Dykxhoorn DM, St Pierre R, Linn T. A set of compatible tac promoter expression vectors. Gene. 1996; 177: 133-6.
